# Mucocutaneous Leishmaniasis: clinical markers in presumptive diagnosis

**DOI:** 10.1590/S1808-86942011000300018

**Published:** 2015-10-19

**Authors:** João Luiz Cioglia Pereira Diniz, Manoel Otávio da Rocha Costa, Denise Utsch Gonçalves

**Affiliations:** 1 MD. ENT; MSc student of Infectology and Tropical Medicine; 2 PhD; Professor at the Graduate Studies Program in Health Sciences: Infectology and Tropical Medicine - UFMG; 3 PhD; Professor at the Graduate Studies Program in Health Sciences: Infectology and Tropical Medicine of the Federal University of Minas Gerais - UFMG

**Keywords:** diagnosis, leishmaniasis, mucocutaneous, otolaryngology

## Abstract

Mucocutaneous Leishmaniasis (ML) can lead to serious sequela; however, early diagnosis can prevent complications.

**Aim:**

To evaluate clinical markers for the early diagnosis of ML.

**Materials and Methods:**

A series study of 21 cases of ML, which were evaluated through clinical interview, nasal endoscopy, biopsy and the Montenegro test.

**Results:**

A skin scar and previous diagnosis of cutaneous leishmaniasis (CL) were reported in 8(38%) patients, and 13(62%) of them denied having had previous CL and had no scar. Nasal/oral symptom onset until the ML diagnosis varied from 5 months to 20 years, mean value of 6 years. In the Montenegro test, the average size of the papule was 14.5 mm, which did not correlate with disease duration (*p*=0.87). The nose was the most often involved site and the extension of the injured mucosa did not correlate with disease duration. The parasite was found in 2 (9.52%) biopsy specimens.

**Conclusions:**

ML diagnosis was late. Finding the parasite in the mucosa, cutaneous scar and/or previous diagnosis of CL were not clinical markers for ML. ML diagnosis must be based on the Montenegro test, chronic nasal and/or oral discharge and histological findings ruling out other granulomatous diseases.

## INTRODUCTION

American tegumentary leishmaniasis (ATL) is a severe public health problem in Brazil and throughout the world, as well. It is endemic in 88 countries and it is the second most important disease among those caused by protozoa with medical relevance, second only to malaria. Minas Gerais is the eight Brazilian state in number of reported cases and the first in Southeastern Brazil in terms of number of cases/year. Of the 853 cities in the state, 401 have reported ATL transmission. Between 2001 and 2006, of the 11,007 cases reported in Minas Gerais, there were 100 deaths caused by the disease, which has a well-established treatment and it must be reported to the health authorities^1^.

ATL is a non-contagious infectious disease, caused by different species of protozoa of the *Leishmania* genus, which affects the skin and mucosae. It is primarily a zoonotic infection, involving other animals besides humans, which can be a secondary infestation^2^.

Despite being less frequent than the cutaneous form, the involvement of the nasal and/or oral mucosae is usually more severe, which may leave sequelae and cause death^3^, ^4^. Moreover, the psychosocial stigma arising from this disease is something that has not been measured yet by health surveillance services, since only those who suffer their consequences can feel them, not much for the physical pain, but rather because of the psychological, social and behavioral consequences brought about by this often repulsive characteristic of the disease, affecting those who develop advanced and mutilating forms of the disease^1^. Development time is the biological factor which determines the parasite spread and it is one of the main causes of the mucosal lesions severity and their consequences^3^, ^4^.

The ATL incubation period varies substantially. Infection in closed populations when the inoculation time is identified shows an incubation period of ten to 60 day^5^. The cutaneous form of the disease is the predominant one in 95% of the reported cases, followed by the mucosal type in 3%-5% of the cases^1^.

Mucosal leishmaniasis (ML) is a form of tegumentary leishmaniasis associated with *L. braziliensis, L. panamensis* and, less frequently, *L. amazonensis*. In most of the cases, the mucosal disease happens after the skin lesions, and the diagnosis of the mucosal involvement is established only months to years after the clinical cure of the initial skin infection site. Some patients may have nasal involvement without skin disease^6^. The goal of the present study is to assess the clinical markers which may have an impact on the early diagnosis of mucosal leishmaniasis.

## MATERIALS AND METHODS

The Reference Ward for Mucosal Leishmaniasis of the ENT Annex of our University Hospital has received an average of two new cases per month of nasal lesions suspected of being mucosal leishmaniasis, coming from the municipal health care network. We carried out a cross-sectional study between April of 2008 and April of 2009, when all the patients seen in the ward were assessed by a structured interview, ENT exam with nasal endoscopy and biopsy of the lesion, when indicated, and followed by an initial period of 12 months. As inclusion criterion, we used a referral with suspicion of mucosal leishmaniasis in patients who had not been previously treated for cutaneous leishmaniasis. Patients with confirmed diagnosis of other diseases involving the upper respiratory tract mucosa were taken off the study.

The proposed protocol for each patient included the following: 1) clinical interview to assess gender, age, report of a previous skin lesion, onset of the first symptoms on mucosae (in months), mucosal lesion location (nasal and/or oropharyngeal); 2) nasal endoscopy specifying the sites involved; 3) biopsy of the suspected mucosal lesions; 4) results from the Montenegro test. Confirmed cases were referred to the Reference Center for Infectious and Parasitic Diseases of the institution for clinical treatment.

This project was approved by the Ethics in Research Committee of the Institution under registration # 292/07. The patients were informed about the objectives, methodology and possible risks and benefits of the study, and they all signed the informed consent form.

## RESULTS

We studied 21 patients with mean age of 59 years, standard deviation of 13 years; of these, seven (35%) were women and 14 (65%) were men. As far as a past of skin lesion is concerned, eight (38%) patients reported prior skin lesion and had scars, while 13 (62%) did not remember and did not have any scar from previous skin involvement.

Time of nasal and/or oropharyngeal symptoms onset until diagnostic definition varied between five months and 20 years, with mean duration of six years. Table 1 shows data from the 21 patients investigated.

**Table 1:** Patients with recent diagnosis of mucosal leishmaniasis seen in the Otorhinolaryngology clinic of our University Hospital, between April of 2008 and April of 2009.

Patient	Gender	Age (years)	Skin lesion	Mucosal symptom onset (months)	Montenegro test (mm)	Mucosal lesion location	Biopsy (parasite present)
01	F	72	Yes	24	10	Nasal/Oropharyn	No
02	M	64	No	120	8	Nasal	No
03	F	75	No	17	18	Nasal	No
04	F	46	Yes	120	22	Nasal	No
05	M	69	No	-	10	Nasal	No
06	M	72	No	24	5	Nasal/Oropharyn	No
07	M	56	No	5	13	Nasal/Oropharyn	No
08	M	58	No	18	Positive^a^	Nasal/Oropharyn	No
09	F	74	No	240	Positive^a^	Nasal/Oropharyn	No
10	M	56	No	108	17	Nasal	No
11	M	74	Yes	24	Positive^a^	Nasal	Yes
12	M	47	Yes	60	Positive^a^	Nasal	No
13	M	77	Yes	24	10	Nasal/Oropharyn	No
14	M	72	No	24	20	Nasal/Oropharyn	No
15	M	45	No	132	25	Nasal/Oropharyn	No
16	M	41	Yes	24	Positive^a^	Nasal/Oropharyn	Yes
17	M	24	Yes	4	15	Nasal	No
18	F	59	No	-	Positive^a^	Nasal	No
19	F	73	No	12	Positive^a^	Oropharynx	No
20	M	34	Yes	11	Positive^a^	Nasal/Oropharyn	No
21	F	47	No	240	8	Nasal	No

a Positive between 10 and 20 mm

**Graph 1:**
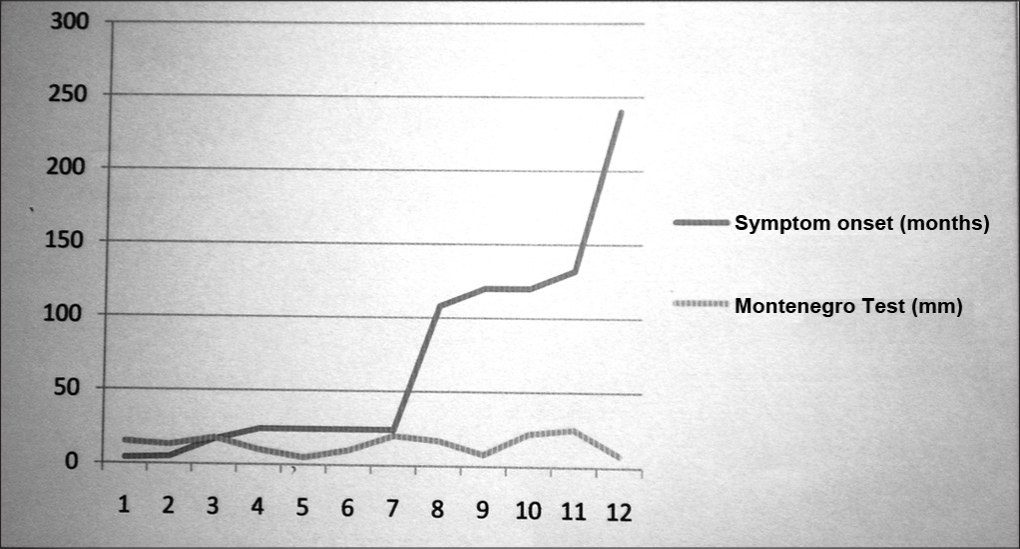
Correlation between the Montenegro test and symptom onset - Papule diameter in the Montenegro skin test and clinical symptom evolution time of the mucosal leishmaniasis of patients seen in the Otorhinolaryngology Clinic of the University Hospital between April 2008 and April of 2009.

The Montenegro test showed a mean papule of 14.5 mm (SD= 6.8 mm), which was not correlated to disease duration (*p*=0.87, Graph 1).

As to the involvement site, nasal involvement alone happened to 10 (47%) cases, simultaneous involvement of the nose and oropharynx happened to 10 (47%) and the oropharynx alone was involved in one (4.8%) case. We did not observe correlation between involvement site and the time of symptom onset (*p*=0.14). We carried out a histological assessment of the lesion in 21 patients, and the parasite was found in two (9.5%) of the nasal or oral mucosae evaluated. Table 1 depicts a summary of the data analyzed.

## DISCUSSION

The nasal mucosa is a preferred site for the lesions caused by *L. braziliensis*, although the oral mucosa, pharynx and larynx may also be affected^4^. Of the 21 patients examined, 20 (94%) had nasal lesions alone, or associated with oropharyngeal lesions. The earliest signs and symptoms of mucosal leishmaniasis are nasal obstruction, epistaxis - arising from granuloma formation, in a few days or months, septum perforation may ensue^6^.

A past of ATL or a typical skin scar was seen in most of the patients assessed. For mucosal leishmaniasis diagnosis, clinical history and the typical skin scar have been considered important clinical markers to corroborate the diagnosis of ML in patients with non-specific granulomatous nasal/oral lesions^7^. In the present study, a past of skin lesion or scan indicating skin leishmaniasis did not prove to be a good marker to forecast the diagnosis of mucosal leishmaniasis. In this series, 62% of the patients did not remember whether or not they had had skin involvement and did not have the suggestive scar of a past lesion. This is an important piece of information because it reflects the limited value of considering the negative history of the patient as to prior leishmaniasis or the lack of a scar as indicators not to consider the diagnostic hypothesis of a mucosal leishmaniasis in a patient with chronic rhinitis without a definite diagnosis. Since it is not very common to find the parasite in the skin lesion^5^, ^7^, confirmed by the present study, what we are left with are the immune tests to help define the mucosal leishmaniasis.

The early mucosal leishmaniasis diagnosis is the main challenge of the Leishmaniasis Tegumentary Americana Surveillance Program of the Health Department aiming at reducing the deformities caused by the disease^2^.

The long time passed between symptom onset and the etiological diagnosis of the mucosal form of ATL may reflect the limitation in the training of most physicians concerning a proper approach for mucosal leishmaniasis, the general practitioner - often times not trained to properly approach nasal complaints, or the specialist, who frequently only does a nasal biopsy, which, in the case of mucosal leishmaniasis, is non-conclusive in most of the cases^5^, ^7^.

Diagnostic delays, which mean time in the present study was of six years, could be justified by the fact that the patients did not seek medical care. Nonetheless, since chronic nasal obstruction is the complaint which has a direct impact on the quality of life and the work capacity of the individual, it is improbable that such delay has been caused by a delay in seeking medical care^8^. In fact, LM patients report having been treated for chronic rhinitis during long periods of time, without a definitive etiological diagnosis^6^. In a study carried out in the state of Paraná, Silveira et al. reported that the time interval between the skin lesion and the nasopharyngeal involvement was of up to two years in 30.4% of the patients, and higher than ten years in 50.0%^9^.

The Montenegro skin test is the most broadly used and reliable to screen individuals with suspicion of ATL, considering the fact that the test has over 90% of sensitivity and specificity, as per shown in different studies^4^, ^10^, ^11^, ^12^. In endemic areas, the positive Montenegro test can be interpreted as previous leishmaniasis, previous injection of the antigen used in the test, exposure to the parasite without disease (infection), allergy to the test diluting agent or crossed reaction with other diseases (Chagas, sporotrichosis, Virchowian leprosy, tuberculosis, chromomycosis, and others)^2^. As it happens to any other screening test, result validity will depend on clinical history and disease prevalence in the population being studied^2^. The mean papule diameter in the Montenegro test found in the present series is in agreement with literature data^13^, just like the lack of correlations between the papule diameter and the time of symptom onset.

The search for antibodies by indirect immunofluorescence must not be utilized as the sole criterion for the diagnosis of ATL, it may be associated with other diseases of the differential diagnosis to the Montenegro test results or to other parasitic techniques^2^. Patients with mucosal lesions have higher and more persistent titers of this reaction, which can be useful to follow up the response to treatment in the mucosal form of the disease^2^, ^9^. Bearing in mind that *leishmania sp* is a protozoa which stimulates cell immunity, we can better understand the reason why the formation of antibodies is limited, when assessed by the immunofluorescence method^2^, ^11^, ^14^.

The Polymerase Chain Reaction (PCR) is a useful tool in the diagnosis of ATL, considering the difficulties in isolating the parasites from the mucosal lesions, especially when the goal is to identify the species^15^. The reaction has been utilized for research purposes, and it is not very much employed in routine diagnostic approaches^2^.

ATL's differential diagnoses includes paracoccidioidomycosis, epidermoid carcinoma, basal cell carcinoma, lymphomas, rhinophyma, rhinosporidiosis, entomophthoromycosis, Virchowian leprosy, tertiary syphilis, traumatic septal perforation or drug use, allergic rhinitis, sinusitis, sarcoidosis, Wegener Granulomatosis, among other rarer diseases^7^, ^14^.

ATL is a disease of mandatory reporting to health authorities. All confirmed cases must be reported and investigated by health care services by means of the Investigation Form standardized by the Notification Severity Information System - Sistema de Informaçã o de Agravos de Notificaçã o (SINAN)^2^. The information system follow up and evaluation must be under the responsibility of the technical department responsible for the ATL surveillance in the three levels of public administration: city, state and federal^2^.

Social and cultural determining factors may impact notification. In the study carried out in Acre, and endemic area with active public policies to control leishmaniasis, it was reported that in the micro region where there was a higher incidence of the mucosal form of the disease (41.0%), the time interval between symptom onset and medical care varied between 2 and 9 months^16^. The supposed higher occurrence of the mucosal form would probably be the result of an active search for suspicious cases and an efficient reporting system.

Actions geared towards early diagnosis and proper treatment of ATL cases must be the responsibility of municipal secretariats of health, with the support of the state secretariats and that from the Federal Health Department. Patient care may be carried out by means of spontaneous demand at the health care centers, by active search of cases in transmission areas - when indicated by epidemiological surveillance, or by the family health care team; or, still, in risky areas where it is difficult for the population to have access to health care centers^2^.

## CONCLUSION

In conclusion, the late diagnosis of mucosal leishmaniasis seems to be associated with educational, social, economic and geographic factors. Otorhinolaryngological exam associated with the Montenegro test continue to be the most important elements for the diagnosis, although usually of presumptive character. We need further studies using new techniques aiming at identifying the parasite or its components in the mucosal lesions in order to improve diagnostic accuracy.

As far as primary health care is concerned, it is worth stressing the need to properly train Family Health Care Teams to actively search cases in endemic areas, as well as to appreciate the complaints of these patients. It is probable that a greater availability of the Montenegro test and an encouragement concerning continued medical education are essential conditions for an early diagnosis of ATL.
